# LSD-YOLO: Enhanced YOLOv8n Algorithm for Efficient Detection of Lemon Surface Diseases

**DOI:** 10.3390/plants13152069

**Published:** 2024-07-26

**Authors:** Shuyang Wang, Qianjun Li, Tao Yang, Zhenghao Li, Dan Bai, Chenwei Tang, Haibo Pu

**Affiliations:** 1 College of Information Engineering, Sichuan Agricultural University, Ya’an 625000, China; wangshuyang@stu.sicau.edu.cn (S.W.); liqianjun@stu.sicau.edu.cn (Q.L.); 202106041@stu.sicau.edu.cn (T.Y.); 202106047@stu.sicau.edu.cn (Z.L.); 202205579@stu.sicau.edu.cn (D.B.); 2 College of Computer Science, Sichuan University, Chengdu 610065, China; tangchenwei@scu.edu.cn; 3 Ya’an Digital Agricultural Engineering Technology Research Center, Ya’an 625000, China

**Keywords:** lemon disease, YOLOv8, small objects, attention mechanisms, object detection

## Abstract

Lemon, as an important cash crop with rich nutritional value, holds significant cultivation importance and market demand worldwide. However, lemon diseases seriously impact the quality and yield of lemons, necessitating their early detection for effective control. This paper addresses this need by collecting a dataset of lemon diseases, consisting of 726 images captured under varying light levels, growth stages, shooting distances and disease conditions. Through cropping high-resolution images, the dataset is expanded to 2022 images, comprising 4441 healthy lemons and 718 diseased lemons, with approximately 1–6 targets per image. Then, we propose a novel model lemon surface disease YOLO (LSD-YOLO), which integrates Switchable Atrous Convolution (SAConv) and Convolutional Block Attention Module (CBAM), along with the design of C2f-SAC and the addition of a small-target detection layer to enhance the extraction of key features and the fusion of features at different scales. The experimental results demonstrate that the proposed LSD-YOLO achieves an accuracy of 90.62% on the collected datasets, with mAP@50–95 reaching 80.84%. Compared with the original YOLOv8n model, both mAP@50 and mAP@50–95 metrics are enhanced. Therefore, the LSD-YOLO model proposed in this study provides a more accurate recognition of healthy and diseased lemons, contributing effectively to solving the lemon disease detection problem.

## 1. Introduction

The lemon, a small evergreen tree of the genus Citrus in the Rutaceae family, is prized for its tart flavor and distinctive refreshing aroma, making it a popular ingredient in cooking, flavoring, and beverage preparation. Lemons are rich in vitamin C, citric acid, and high levels of potassium [1], all of which provide significant health benefits. Additionally, lemons are reputed to have some medicinal properties [2] and are used in the treatment colds [3], as well as in the prevention and management of hypertension and symptoms of myocardial infarction. Lemon trees exhibit rapid growth and thrive in warm climates. By 2022, the area dedicated to lemon cultivation in China reached 125,070 hectares, yielding a production of 2,697,500 tons. However, the expansion of lemon cultivation has been accompanied by an increased incidence of pests and diseases, significantly impacting the quality and yield of lemons [4]. The manual identification of diseased lemons for subsequent control is labor-intensive and resource-consuming and is prone to errors and omissions, making it inadequate for large-scale farmland management. Fortunately, advancements in artificial intelligence offer an effective solution to this problem [5].

With the rapid development of deep learning techniques, numerous target detectors have been proposed, including Faster RCNN [6], YOLO [7], SSD [8], RetinaNet [9]. Many of these detectors have been effectively applied to the detection of plant diseases. For instance, Kundu et al. effectively improved disease detection accuracy by using the k-means algorithm to extract regions of interest, thus reducing interference from irrelevant features [10]. Gangwar et al. integrated the VGG16, VIT [11], and CvT [12] models, simplifying the depth of transformer blocks, which reduced both training time and storage space requirements [13]. Yin et al. [14] incorporated deformable convolutions into the SSD network to improve the representation of target detail features and the identification of weak information, achieving an average accuracy (mAP@50) of 97.1%. Li et al. [15] integrated SENet [16] and ResNeXt [17] into YOLOv3 [18] to design a model with reduced computational complexity and enhanced feature extraction capability, resulting in an average accuracy of 96.28%, with a detection speed of 106 frames per second. Zhang et al. [19] replaced the backbone of the YOLOv4 [20] model with MobileNet-V2 [21] and integrated a three-layer Bi- FPN [22] to ensure the creation of a lightweight model, achieving an overall accuracy of 89%. By applying deep learning to plant diseases, not only can the health and yield of crops be effectively improved, but it also helps to transform agricultural resource management, achieving agricultural sustainability [23].

In recent years, both domestic and international scholars have made significant contributions to the task of detecting diseases of various fruits by exploring and optimizing methods from different perspectives, such as feature extraction and neural network architectures. Despite this progress, relatively little research has been dedicated to the specific task of lemon disease recognition. Therefore, further research and investment are needed to address the challenges posed by lemon diseases and to support the lemon industry effectively. The study of lemon and its disease identification face challenges due to factors such as variations in different growth stages, lighting conditions, and random shading. Additionally, the varying degrees of disease severity contribute to the diversity and complexity of affected areas, complicating the accurate identification of lemons and their diseases. To address the above challenges, we collected 726 relevant images and then cropped the high-resolution images to finally obtain a total of 2022 images. Subsequently, this study utilized the YOLOv8 [24] target detector, which ultimately led to the proposal of a new LSD-YOLO. This model incorporates the CBAM attention mechanism [25] and the SAConv convolutional block [26], alongside an additional small-target detection layer to enhance the detection performance. The experimental results demonstrate that the improved algorithm more accurately localizes healthy and diseased lemons and adapts more effectively to complex natural environments.

## 2. Related Work

### 2.1. Attentional Mechanisms

Attentional mechanisms have become pivotal in enhancing the performance of deep learning models by enabling dynamic focus on the most relevant parts of the input data. In the domain of computer vision, attention mechanisms have been extensively applied to improve the accuracy and efficiency of target detection tasks. For instance, the Convolutional Block Attention Module (CBAM) proposed by Woo et al. [25] integrates spatial and channel attention to refine feature representations and has demonstrated significant improvements in various vision tasks. Similarly, the work of Hu et al. [16] on squeeze-and-excitation networks introduced channel-wise attention, which adaptively recalibrates channel-wise feature responses and has been influential in improving model performance.

In the context of object detection, attention mechanisms have been integrated into several state-of-the-art models. Lin et al. [9] introduced the RetinaNet, which utilizes a focal loss function to handle class imbalance, while subsequent works have incorporated attention modules to further enhance feature discrimination. Zhang et al. [19] enhanced the YOLOv4 model with MobileNet-V2 and Bi-FPN, demonstrating the utility of attention in lightweight models for maintaining high accuracy.

The Transformer [27] model by Vaswani et al. has also significantly influenced the use of attention in computer vision. Dosovitskiy et al. extended the Transformer architecture to vision tasks, resulting in the Vision Transformer (ViT) [11], which employs self-attention to process image patches and has achieved competitive performance on image classification benchmarks.

These advancements highlight the critical role of attentional mechanisms in modern computer vision applications, underscoring their ability to improve feature extraction, enhance model interpretability, and increase overall detection accuracy. In this work, we integrated the CBAM into our model to help the network to emphasize critical features. 

### 2.2. Small Object Detection

The detection of small objects is a critical challenge in computer vision, primarily due to their low resolution, low contrast, and small size. These characteristics make it difficult for standard object detection algorithms to accurately identify and localize small objects. These factors often hinder accurate detection in images. To address this, researchers have developed various methods to improve the detection performance for small objects.

One such technique is feature pyramid networks (FPNs) [28], introduced by Lin et al., which leverage a pyramid structure to create high-level semantic feature maps at different scales. This allows the detection of objects of varying sizes, including small objects, by combining feature maps from different layers. Another significant contribution is the single-shot multibox detector (SSD) by Liu et al., which incorporates multi-scale feature maps and default boxes of different aspect ratios into its framework. The SSD can detect objects at multiple scales, improving the detection accuracy for smaller objects.

Regarding the YOLO series of models, YOLOv3 introduced a multi-scale detection head, which helps in detecting small objects by combining information from different resolutions. Subsequent versions of YOLOv3 have also incorporated strategies for better small object detection.

Attention mechanisms, such as the CBAM, have been applied to emphasize critical features and suppress irrelevant ones. This helps models focus more on small objects within an image. Similarly, the use of channel and spatial attention mechanisms has been shown to improve the detection accuracy for small objects by dynamically adjusting the importance of feature maps.

### 2.3. Switchable Atrous Convolution

Ordinary convolution operations extract local features in an image by performing sliding operations on the input data through a convolution kernel. This approach has made great progress in tasks such as image classification and target detection. However, it also presents challenges, particularly with a fixed receptive field size, which may limit the model’s ability to capture information at different scales in the input data. Local connectivity may also cause the model to ignore global information, adversely affecting the performance. In contrast, Switchable Atrous Convolution (SAConv) can significantly improve the performance of object detection by applying different atrous rate for convolution. Furthermore, it incorporates a global context module and a new weight locking mechanism, which improve the model’s ability to capture and utilize information from both local and global contexts.

**Figure 1:**
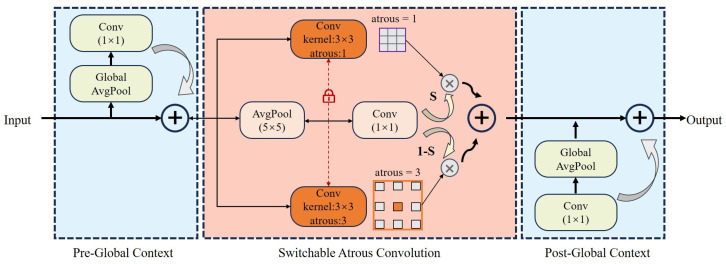
Architecture of SAConv with SAC components, locks, and global context module.

The architecture of SAC (Switchable Atrous Convolution) consists of three main parts: the SAC component and two global context modules positioned before and after it. The SAConv structure is shown in Figure 1. These modules help the network to adaptively adjust the sensing field according to the changes in the scale and position of the target in the image, capturing the global information of the overall image and enabling the SAC component to work efficiently in a wider range of contexts.

SAC employs a varying null rate for the convolution operation when processing the same input features. Null convolution effectively extends the receptive field by introducing extra space in the convolution kernel, together with a switching function to fuse the results of different convolutions, thus making the network more flexible with respect to the size and scale of different features.

Qiao et al. [26] use y=Conv⁡(x;w;r) to denote the convolution operation, with x as the input, y as the output, weights as w, and the atrous rate as r Then, we can convert a convolutional layer to SAC as follows:

(1) $$\begin{array}{c} \begin{array}{c} \mathrm{Conv}(x,w,1)\overset{Convert}{\underset{to\;SAC}{\to }}S(x)\cdot \mathrm{Conv}(x,w,1)+(1-S(x))\cdot \mathrm{Conv}(x,w+\Delta w,r) \end{array} \end{array}$$

where r is the hyperparameter of the SAC, ∆w is the trainable weights, and the switching function S(·) is implemented as an average pooling layer with a kernel of 5 × 5 and a convolutional layer of 1 × 1. This switching allows the convolutional computation to be soft-switched between different atrous rates.

## 3. Materials and Methods

### 3.1. Data Acquisition and Processing

**Figure 2:**
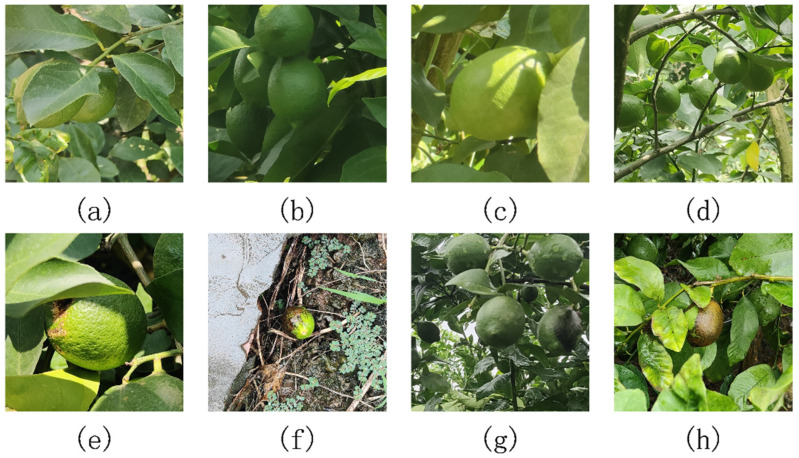
Samples in different shooting environments (**a**) leaf shade, (**b**) poor lighting, (**c**) single lemon, (**d**) multiple lemons, (**e**) shade present in diseased lemons, (**f**) different backgrounds, (**g**) diseased lemons and healthy lemons in the same frame, and (**h**) good lighting.

In this study, we conducted fieldwork in the renowned lemon-producing region of Anyue County, Ziyang City, Sichuan Province. Over the fruiting season of lemons, spanning from September to November in 2023, a comprehensive collection comprising 726 images of lemons and diseased specimens was amassed. These images were captured under varying weather conditions, including sunny and lightly raining environments. Each image encapsulated diverse lighting scenarios, including front light, back light, and incidental shadows, with variations in angles and distances. The dataset comprised 424 close-up shots and 302 high-resolution images from varying distances. Then, we crop the high-resolution images, by finding the location of the target, and then crop 1024 × 1024-sized images from the original images and re-generate the annotation data, and the edge coordinate information generated by the cropping process is also preserved. The 726 original images were finally expanded to 2022 cropped images. The augmentation strategy not only improves the richness of the dataset but also mitigates potential overfitting risks, thereby enhancing the model’s performance and generalization capabilities. Figure 2 shows some samples of healthy and diseased lemon datasets under different conditions.

Before the data can be subjected to the target detection algorithm, the targets need to be labeled. In this process, we divided the dataset into two categories of lemon data according to whether the target was diseased or not (healthy lemons and diseased lemons), and the labeling information focused on the category of the target, as well as the location of the center point and the length and width. The labeling process was performed using the cvat tool, which then generated the dataset format in the YOLO format. In order to better evaluate the model’s performance and improve the generalization ability and stability of the model, we use a five-fold cross-validation [29] approach, which ensures that each sample can be present in both the training and validation sets by dividing the dataset into five subsets, using four of these subsets for training at a time and then validating the dataset on one of the remaining subsets. The data distribution between these sets is shown in Table 1.

### 3.2. The Proposed LSD-YOLO Model

**Figure 3:**
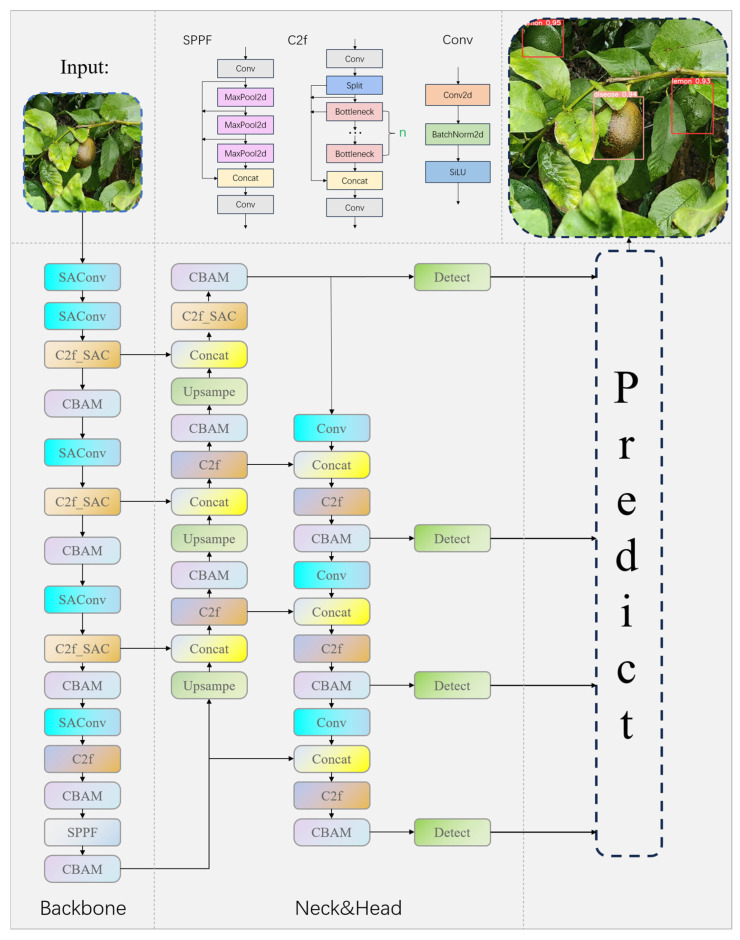
Network structure of LSD-YOLO.

In reality, diseased lemons present dynamic and complex characteristics such as color depth, shape and size due to different degrees of disease, which, together with problems such as shading and the angle of lighting in a real environment, add considerable difficulty to identify diseased lemons more accurately [30]. At the same time, the color similarity between normal lemons and leaves, random occlusion and other issues are also a challenging problem. At the same time, the YOLOv8 downsampling multiplier is relatively large, which makes the small target lemon and disease area small; the characteristics of disease spot dispersion are more difficult to be learned in a more top-level module. To overcome the above problems, we proposed the LSD-YOLO model based on YOLOv8. Based on YOLOv8, we introduced SAConv in the backbone network and designed C2f-SAC, which allowed the model to adapt to features of different scales more flexibly. And the CBAM attention mechanism is added to let the model focus on important features and suppress unnecessary features. For small targets, we add extra detection heads to improve the performance of recognizing small targets. The LSD-YOLO structure proposed in this paper is shown in Figure 3.

**Figure 4:**
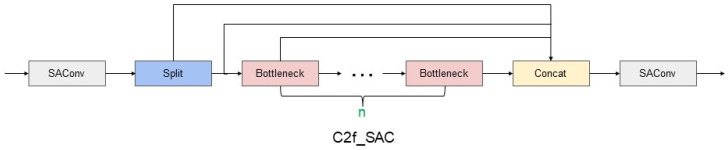
Structure of the C2f_SAC module.

SAConv can make the network more flexible in adapting to features of different scales to recognize objects in images more accurately. The SAC component and global context module occupied by SAConv can well optimize the fixed sensory field and local continuity problems brought by ordinary convolution. Therefore, we incorporate it into the backbone of YOLOv8, and also incorporate SAConv into C2f to get the C2f_SAC module, the specific structure of C2f_SAC module is shown in Figure 4. In C2f_SAC, the input of the previous layer, after passing through a SAConv, can capture the information in the input feature maps in different scales, and dynamically fuses the convolution results at these different atrous rate. At the same time, while keeping the feature map size constant, it can capture the feature information at different scales and integrate the global information efficiently at the global scale.

To improve the characterization of the network, we use the CBAM attention mechanism. It is not only a lightweight module, but it also takes into account both channel and spatial dimensions. The channel dimension focuses on “categories”, while the spatial dimension focuses on “locations”, and these two dimensions complement each other. We set the ratio parameter to a small value of 8, which increases the impact of the average pooling operation and makes the model more concerned with global information. Meanwhile, considering that the stacking of blocks of the same shape, as pointed out by VGGNet [31], can obtain a fairer result, we set the convolution in the spatial attention module to 3 × 3 convolution. The detailed operation is as follows.

(2) $$\begin{array}{cc} M_{s}\left(F\right) & \;=\sigma \left(f^{3\times 3}\left(\left[AvgPool\left(F\right);MaxPool\left(F\right)\right]\right)\right)\\ & \;=\sigma \left(f^{3\times 3}\left(\left[F_{\mathrm{avg}\;}^{s};F_{max}^{s}\right]\right)\right) \end{array}$$

where *σ* denotes the sigmoid function and f 3 × 3 denotes a convolution operation with a convolution kernel size of 3 × 3. $F_{\mathrm{avg}\;}^{s}$; $F_{max}^{s}$ denote the average pooling feature and the maximum pooling feature, respectively.

The FPN + PAN [32] method used in YOLOv8 fuses feature maps of multiple scales together to obtain feature maps of 80 × 80 × 256, 40 × 40 × 516, 20 × 20 × 1024. And we fused more shallow features 160 × 160 × 64 on this basis; this shallow feature map has more detailed information, which helps to better detect and localize small targets. At the same time, the shallower feature maps are stitched together with the deeper feature maps, which effectively retains more detailed information in the shallow feature maps while also capturing the global context and abstract features of the objects in the deeper features. A new 160 × 160 × 128 feature map is finally obtained, which leads to a new detection head to improve the detection performance of the model for small targets.

### 3.3. Evaluation Indicators for the Model

The experimental environment of this paper was conducted using Python 3.10, CUDA 12.1, and the PyTorch 2.2.1 deep learning development framework. The computer used in the study is equipped with Intel(R) Core(TM) i9-14900K (Intel Corporation, santa Clara, CA, USA), 24 core CPU and NVIDIA GeForce RTX 090 GPU, running on the Ubuntu 22.04 operating system.

In this study, the relevant hyperparameters are as follows: a 640 × 640 pixel image is taken as the same input, and AdamW [33] is used as the optimizer, with an initial learning rate of 0.001667, a momentum of 0.9, and a weight decay value of 0.0005. After considering the memory requirement, convergence speed, generalization ability, this paper sets the batch size during training to 8 and the number of iterations for training the same set to 200 rounds.

In order to accurately measure the performance of the algorithm in different aspects, we choose a series of classical performance metrics, including Precision, Recall, mean average precision (mAP), overall standard deviation (σ), etc., to measure the performance of the algorithm. Among them, Precision and Recall are used to measure the precision and coverage of the algorithm. Precision indicates the ratio between the number of targets correctly detected by the model and the number of all detected targets, while Recall indicates the ratio between the number of targets correctly detected by the model and the number of all targets in the dataset. mAP is used to assess the performance of the algorithm on different categories and is a comprehensive metric. The overall standard deviation assesses the stability of the model. The formulas for these metrics are as follows.

(3) $$Precision=\frac{TP}{TP+FP}$$

(4) $$Recall\;=\frac{TP}{TP+FN}$$

(5) $$AP=\int_{0}^{1}P\left(R\right)dR$$

(6) $$mAP=\frac{{AP}_{1}+{AP}_{2}+\cdots +{AP}_{n}}{n}$$

(7) $$\mu =\frac{1}{5}\sum_{i=1}^{5}x_{i}$$

(8) $$\sigma =\sqrt{\frac{\sum_{i=1}^{5}{\left(x_{i}-\mu \right)}^{2}}{5}}$$

where TP represents the number of correctly identified positive samples, FP is the number of negative samples incorrectly identified as positive samples, and FN is the number of positive samples incorrectly identified as negative samples. n represents the number of categories, $x_{i}$ is the data for the i-th indicator, μ is the mean value of the indicator, and σ is the overall standard deviation.

## 4. Results and Discussion

### 4.1. Comparison Experiment

To verify the accuracy, validity, and stability of LSD-YOLO, we compared LSD-YOLO with YOLOv8n, YOLOv7-tiny [34], YOLOv5n6, and YOLOv5n [35], which are advanced target detection methods. Table 2 and Table 3 present the Precision (P), Recall (R), mAP@50, mAP@50–95, overall standard deviation, and parameter count discerned by these various models.

**Figure 5:**
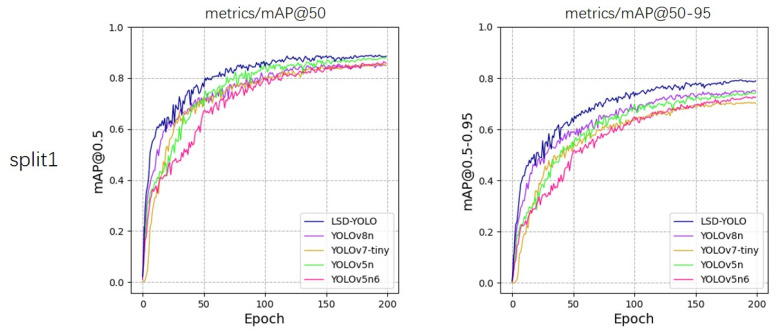
Variations in accuracy for mAP@50 and mAP@50–95 during the training process of five models, evaluated using 5-fold cross-validation on split1.

**Figure 6:**
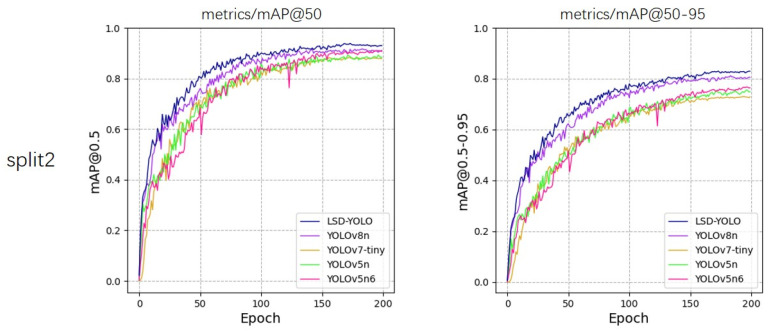
Variations in accuracy for mAP@50 and mAP@50–95 during the training process of five models, evaluated using 5-fold cross-validation on split2.

**Figure 7:**
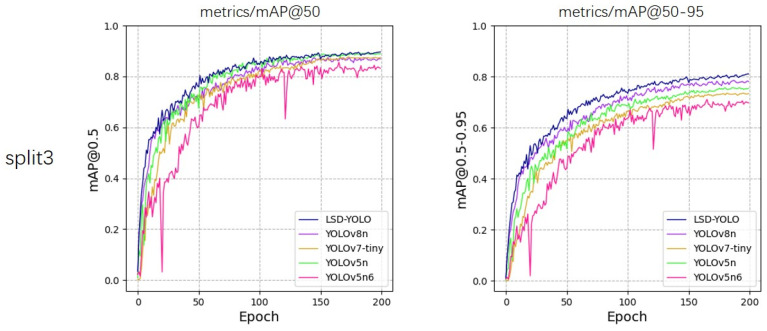
Variations in accuracy for mAP@50 and mAP@50–95 during the training process of five models, evaluated using 5-fold cross-validation on split3.

**Figure 8:**
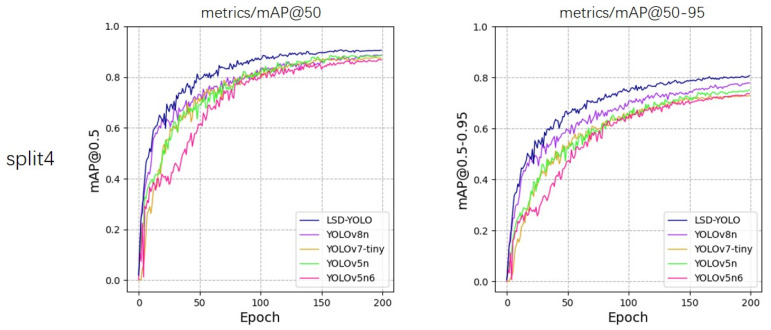
Variations in accuracy for mAP@50 and mAP@50–95 during the training process of five models, evaluated using 5-fold cross-validation on split4.

**Figure 9:**
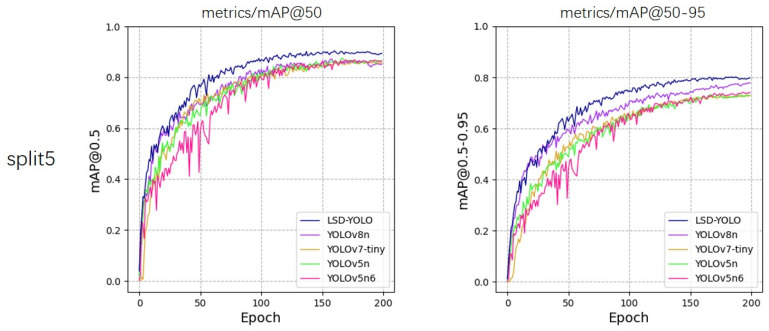
Variations in accuracy for mAP@50 and mAP@50–95 during the training process of five models, evaluated using 5-fold cross-validation on split5.

From the experimental results, our LSD-YOLO has better accuracy, completeness and precision in lemon detection, with mAP@50 reaching 92.89% and 88.36% for healthy lemons and diseased lemons, respectively, as well as higher scores for other metrics. Compared with the original YOLOv8n algorithm, LSD-YOLO reached 90.62% for mAP@50, an improvement of 2.64%, and 80.84% for mAP@50–95, an improvement of 2.97%, with only a 0.34 M parameter increase, which is significantly higher than the detection accuracy of other models. Moreover, the overall standard deviation of the improved LSD-YOLO is smaller than that of the original algorithm for mAP@50 and mAP@50–95, which fully demonstrates the better stability and robustness of the present model. The fluctuations in accuracy across the five models during the training process are depicted in Figure 5, Figure 6, Figure 7, Figure 8 and Figure 9.

### 4.2. Visualization

**Figure 10:**
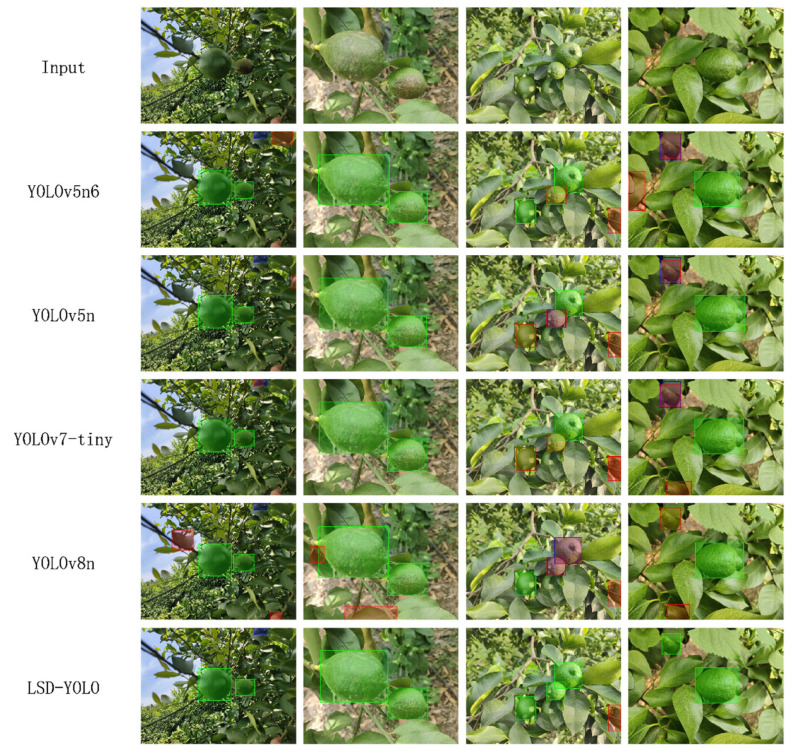
The visualization results of TP, FP, and FN for five models after detection. In these visualizations, green indicates true positives (correctly identified positive samples), blue indicates false negatives (failed to detect positive samples), and red indicates false positives (incorrectly detected as positive samples).

To gain a more intuitive understanding of the model’s performance in detecting lemon diseases in natural environments and to compare the reliability of each model, we visualized the detection results of different models. We selected four images in the validation set of the divided split2 and evaluated them using YOLOv5, YOLOv5n6, YOLOv7-tiny, YOLOv8n, and LSD-YOLO. The visualization results of true positives (TPs), false positives (FPs), and false negatives (FNs) for the five models are shown in Figure 10 below.

From the Figure 10, it is evident that each model exhibits better recognition performance when the target is large and located in the center of the image. However, as the target size decreases, the environmental occlusions and interference from foliage at the edges affect detection accuracy. Under these challenging conditions, the improved LSD-YOLO demonstrates significantly better recognition performance than the other models. This observation confirms that LSD-YOLO offers superior accuracy and reliability.

### 4.3. Ablation Study

In this paper, three methodologies were been employed to enhance the model. To ascertain the efficacy of these methodologies on the model’s performance, ablation experiments were conducted on YOLOv8n. Eight distinct experimental configurations were devised through permutations of various modules. Evaluation metrics including mAP@50, mAP@50–95, the overall standard deviation of the corresponding indicators, and parameters count were employed for assessment. The nomenclature utilized for clarity designates the module dedicated to small object detection as SOD, the Spatial Attention Convolution as SAC, and the Convolutional Block Attention Module as CBAM. Detailed outcomes are presented in Table 4.

Table 4 illustrates that the introduction of the SAConv module leads to notable improvements in mAP@50 and mAP@50–95 by 1.67% and 1.59%, respectively. This underscores the efficacy of incorporating global information and employing convolution with varying atrous rates, with a switching function, to effectively broaden the receptive field. Consequently, the network can capture a more extensive array of contextual information, facilitating efficient feature extraction. Additionally, the integration of the CBAM yields significant performance improvements in the model. Remarkably, the sole introduction of SOD results in a reduction in the overall variance and parameter count for each metric, albeit with a decrease in accuracy. However, the combination of SOD and the CBAM demonstrates effective enhancement. We posit that the initial introduction of SOD, through dimensionality reduction and upgrading operations, leads to information loss. However, the subsequent addition of the CBAM attention mechanism accentuates the compressed low-dimensional features. Subsequent upgrading processes reconstruct and fortify the features, thereby enhancing their expression. In summary, this study contends that the synergy of these three parts constitutes a synergistic approach, resulting in qualitative improvements in detection accuracy with minimal parameter increments while concurrently enhancing model stability.

### 4.4. Applicability

To further investigate the performance and applicability of the model in detecting diseases in other fruits, this study utilized a dataset named “detection1” [36] from Roboflow, specifically focusing on citrus fruits. The dataset comprises 2782 images of citrus fruits, encompassing various degrees of rot, irregular shapes and sizes, and different levels of occlusion. The dataset is categorized into two classes: ‘orange fraiche’ (fresh orange) and ‘orange pourrie’ (rotten orange). We partitioned the dataset in a 7:2:1 ratio, resulting in 1947 images for the training set, 556 for the validation set, and 279 for the test set. Table 5 lists the Precision (P), Recall (R), and mAP@50 of the LSD-YOLO model on this dataset.

As shown in Table 5, the proposed LSD-YOLO model demonstrates commendable performance on the citrus dataset. Specifically, the mAP@50 for fresh oranges and rotten oranges achieved 88.57% and 96.80%, respectively, with an overall average mAP@50 of 92.69%. Additionally, other evaluation metrics also yielded satisfactory results. These findings underscore the enhanced LSD-YOLO model’s applicability and flexibility in detecting diseases across different plant species.

## 5. Conclusions

To address the issue of surface disease during lemon cultivation, this paper proposes the LSD-YOLO model based on the YOLOv8n detector. By integrating the SAConv module and improving the C2f module, we introduce the new C2f-SAC, which can better capture global information and features at different scales. The inclusion of the CBAM attention mechanism and a small-target detection layer further enhances the representation of key features and the detection of small targets, making the model more adept at utilizing and expressing features comprehensively. The experimental results indicate that the proposed LSD-YOLO mAP@50 reaches 90.62% and mAP@50–95 reaches 80.84%. This model demonstrates superior detection accuracy and stability compared to other commonly used objective detection. Compared to the benchmark model, LSD-YOLO can show a better performance in each evaluation metric with the introduction of the 0.34 M parameter number. Therefore, this method proves feasible for lemon surface disease detection, and it can detect healthy and diseased lemons more accurately in natural environments, which provides more powerful technical support for the product quality control of lemons. However, it should not be overlooked that the performance of the model can vary in different environments or with different lemon varieties. Moreover, the difficulty in identifying subtle symptoms makes early disease detection still challenging. In the future work, our study will further optimize the model’s structure, expand the scope of the dataset, especially targeting early-stage disease, to achieve a more accurate identification of diseased lemons, and facilitate the timely detection and treatment of lemon diseases.

## Figures and Tables

**Table 1:** Division of datasets.

	Name	Lemon Instances	Disease Instance	Total
Split1	Training set	3542	591	4133
	Validation set	899	127	1026
Split2	Training set	3525	599	4124
	Validation set	916	119	1035
Split3	Training set	3530	562	4092
	Validation set	911	156	1067
Split4	Training set	3595	577	4172
	Validation set	846	141	987
Split5	Training set	3572	543	4115
	Validation set	869	175	1044

**Table 2:** Comparative analysis of model accuracy metrics at IOU = 0.5.

Model	Lemon (%)	Disease (%)	mAP@50 (%)	$\sigma_{mAP@50}$	Parameter
YOLOv5n	91.47	85.32	88.40	0.73	1.77 M
YOLOv5n6	90.30	84.26	87.28	1.93	3.10 M
YOLOv7-tiny	90.03	83.71	86.87	1.16	6.02 M
YOLOv8n	91.69	84.28	87.98	2.16	3.01 M
LSD-YOLO	92.89	88.36	90.62	1.73	3.35 M

**Table 3:** Comparative analysis of metrics across different models.

Model	Precision (%)	Recall (%)	Lemon (%)	Disease (%)	mAP@50–95 (%)	$\sigma_{mAP@50–95}$
YOLOv5n	87.41	81.76	74.90	74.57	74.73	0.92
YOLOv5n6	85.26	80.14	74.10	73.20	73.65	1.85
YOLOv7-tiny	85.02	80.66	72.57	72.16	72.36	1.02
YOLOv8n	87.54	79.71	78.98	76.77	77.87	1.99
LSD-YOLO	89.22	83.96	80.53	81.15	80.84	1.21

**Table 4:** Results of the ablation study.

Method	mAP@50 (%)	$\sigma_{mAP@50}$	mAP@50–95 (%)	$\sigma_{mAP@50–95}$	Parameter
YOLOv8n	87.98	2.16	77.87	1.99	3.01 M
YOLOv8n + SAC	89.65	1.60	79.46	1.36	3.35 M
YOLOv8n + SOD	87.98	1.88	77.69	1.50	2.93 M
YOLOv8n + CBAM	88.08	2.16	77.95	1.58	3.08 M
YOLOv8n + SAC + SOD	88.98	1.16	78.95	1.02	3.29 M
YOLOv8n + SOD + CBAM	88.25	2.03	78.18	1.76	2.99 M
YOLOv8n + SAC + CBAM	89.53	2.22	79.41	2.48	3.42 M
YOLOv8n + SAC + SOD + CBAM	90.62	1.73	80.84	1.21	3.35 M

**Table 5:** Metrics for different classes at IOU = 0.5.

Classes	P (%)	R (%)	mAP@50 (%)
all	87.92	86.37	92.69
orange fraiche	88.03	78.84	88.57
orange pourrie	87.81	93.89	96.80

## Data Availability

For access to the data from this study, please contact the corresponding author.
